# Passive nonreciprocal transmission and optical bistability based on polarization-independent bound states in the continuum

**DOI:** 10.1515/nanoph-2023-0319

**Published:** 2023-08-08

**Authors:** Shiwen Chen, Yixuan Zeng, Zhongfu Li, Yu Mao, Xiaoyu Dai, Yuanjiang Xiang

**Affiliations:** School of Physics and Electronics, Hunan University, Changsha 410082, China; Ministry of Industry and Information Technology Key Lab of Micro-Nano Optoelectronic Information System, Shenzhen Graduate School, Harbin Institute of Technology, Shenzhen, 518055 P. R. China; College of Physics and Optoelectronic Engineering, Shenzhen University, Shenzhen 518060, China; School of Electronic Information and Electrical Engineering, Hunan University, Changsha 410003, China

**Keywords:** bound states in the continuum, nonreciprocal transmission, optical bistability

## Abstract

Free-space nonreciprocal transmission is a crucial aspect of modern optics devices. The implementation of nonreciprocal optical devices through optical nonlinearity has been demonstrated. However, due to the weak nonlinearity of traditional materials, most self-biased nonreciprocal devices are heavily dependent on the high *Q* strong resonances. In general, these resonances are frequently polarization sensitive. In this work, we propose ultrathin optical metasurface embedding Kerr nonlinearities to achieve nonreciprocal transmission and optical bistability for free-space propagation based on symmetry-protected bound states in the continuum (BICs). Since the structure of the metasurface retains C_4ν_ symmetry, the symmetry-protected BIC is polarization-independent. It is also shown that the nonreciprocal intensity range could be largely tuned by the structure parameters. The demonstrated devices merge the field of nonreciprocity with ultrathin metasurface technologies making this design an exciting prospect for an optical switch, routing, and isolator with optimal performance.

## 1 Introduction

The control of light flow in modern optical devices relies significantly on nonreciprocal transmission [1–5]. However, the manufacture of nonreciprocal electromagnetic devices on traditional platforms is challenging due to the symmetric time-invariant and linear permittivity and permeability tensors. Thus, three physical mechanisms can break reciprocity. Firstly, the application of a dc magnetic bias to magneto-optical materials can obtain the asymmetric permittivity tensor leading to light isolation. Secondly, the time-variant properties of some materials can achieve optical nonreciprocity. Finally, strong electromagnetic nonlinear effects can break reciprocity in different settings, such as using the harmonic generation [6], using phase transitions in VO_2_ layer [7], and nonreciprocal optical nonlinear metasurfaces [8]. Nonlinearity has attractive features and does not require any external bias, making it a promising approach for integration with traditional optical platforms. As light passes through mirror asymmetric devices in opposite directions, the different refractive indices caused by nonlinear materials affects the transmission efficiency of light differently, breaking the reciprocity property. Nonreciprocal transmission by nonlinear metasurfaces methods is based on the Kerr effect, which depends on the local strength of the electric field. Nonreciprocal methods based on nonlinearity have been applied in various systems due to their simplicity and applicability. However, since nonlinear materials typically have weaker optical nonlinearities, these self-biased devices must rely on high-*Q* optical resonance [9].

Bound states in the continuum (BICs) are a unique phenomenon within the continuous spectrum of extended states, exhibiting perfect localization in space and infinite theoretical lifetimes [10–16]. Initially proposed in 1929 by Neumann and Wigner as an electronic system in the context of quantum mechanics, the problem remained a mathematical curiosity for nearly five decades without experimental evidence to support its existence. But now, such interesting, novel, and essential phenomena have been proposed in various wave systems, such as mechanics, electronics, acoustics, and optics. In the field of nanophotonic, the state of optical BIC has been widely discussed over the past few decades due to its high *Q* factor strong resonance. To this day, BICs have been demonstrated to construct in various mechanisms, including symmetry mismatch, parameter tuning, environment engineering, topological charge evolution, parity-time symmetry, and so on. In addition to the well-known symmetry-protected BICs (SP-BICs) developed by the symmetry-restricted out-coupling [17–22], the existence of optical BIC modes can also be classified as the accidental BIC or Fredrich–Wintegen (FW-BIC) [23–25] in brief.

In this letter, we present a novel approach to achieve nonreciprocal transmission using a nonlinear method. We demonstrate that this can be accomplished through the utilization of polarization-independent resonances that are governed by SP-BIC in a metasurface with C_4ν_ symmetry [26]. Generally, the rotational or mirror symmetry of a structure is broken by in-plane [27–29] and out-of-plane symmetry perturbation [30, 31], making resonances excited by SP-BIC polarization-sensitive. However, we report the exciting discovery of polarization-independent SP-BIC in metasurfaces made of equidistantly spaced perturbed high-refractive-index dielectric disks arrays, which retain C_4ν_ features. To further understand the mechanism of resonances, we calculate the scattering powers of different multipoles [32, 33]. Ultimately, we show that a nonlinear metasurface with C_4ν_ symmetry can facilitate free-space nonreciprocal propagation by breaking reciprocity based on polarization-independent SP-BIC. Our findings pave the way for the design and development of new devices with nonreciprocal transmission properties.

## 2 Polarization-independent q-BIC

**Figure 1:**
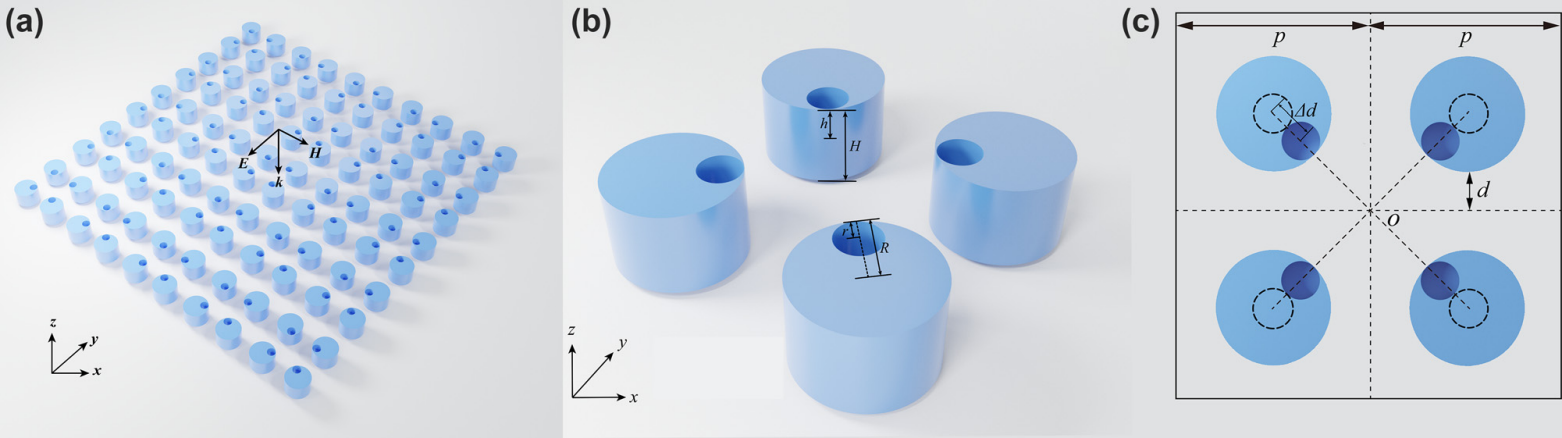
Schematic diagram of the structure. (a) Schematic of the nonreciprocal metasurface: by combining structural asymmetry and material nonlinearity, a monochromatic beam impinging from the up and down sides of the device experiences markedly different transmission levels. (b) Geometry parameters of the supercell consisting of four same square unit. (c) When the etched hole moves towards the center of the circle *O*, the true BIC mode turns to q-BIC mode.

Let us start with a Si metasurface composed of cylindrical meta-atoms with an etched hole; see Figure 1(a). The radii of the cylindrical element atom and the etched cylindrical hole are *R* and *r*, respectively. And the height of cylindrical meta-atoms and etched cylindrical holes are *H* and *h*, respectively. Four cylindrical meta-atoms with the same Geometric parameters form a 2 × 2 square supercell shown in Figure 1(b). We control the asymmetry of the metasurface by adjusting the position of the etched cylindrical holes. When the etched hole and the cylindrical meta-atoms share the same center, the period of the metasurface is *p* = 500 nm. Then we let this etch hole move along the dotted line towards the center *O* displayed in Figure 1(c). We define this asymmetric factor *α* as the distance the etched hole moves toward the center divided by the difference between the radius of the metasurface *R* and etched cylindrical holes *r*, that is Δ*d*/(*R* − *r*). When *α* ≠ 0, the period of the metasurface changes to *a* = 2*p. Obviously, no matter how this asymmetric factor changes, our structure remains C_4ν_ symmetry.

**Figure 2:**
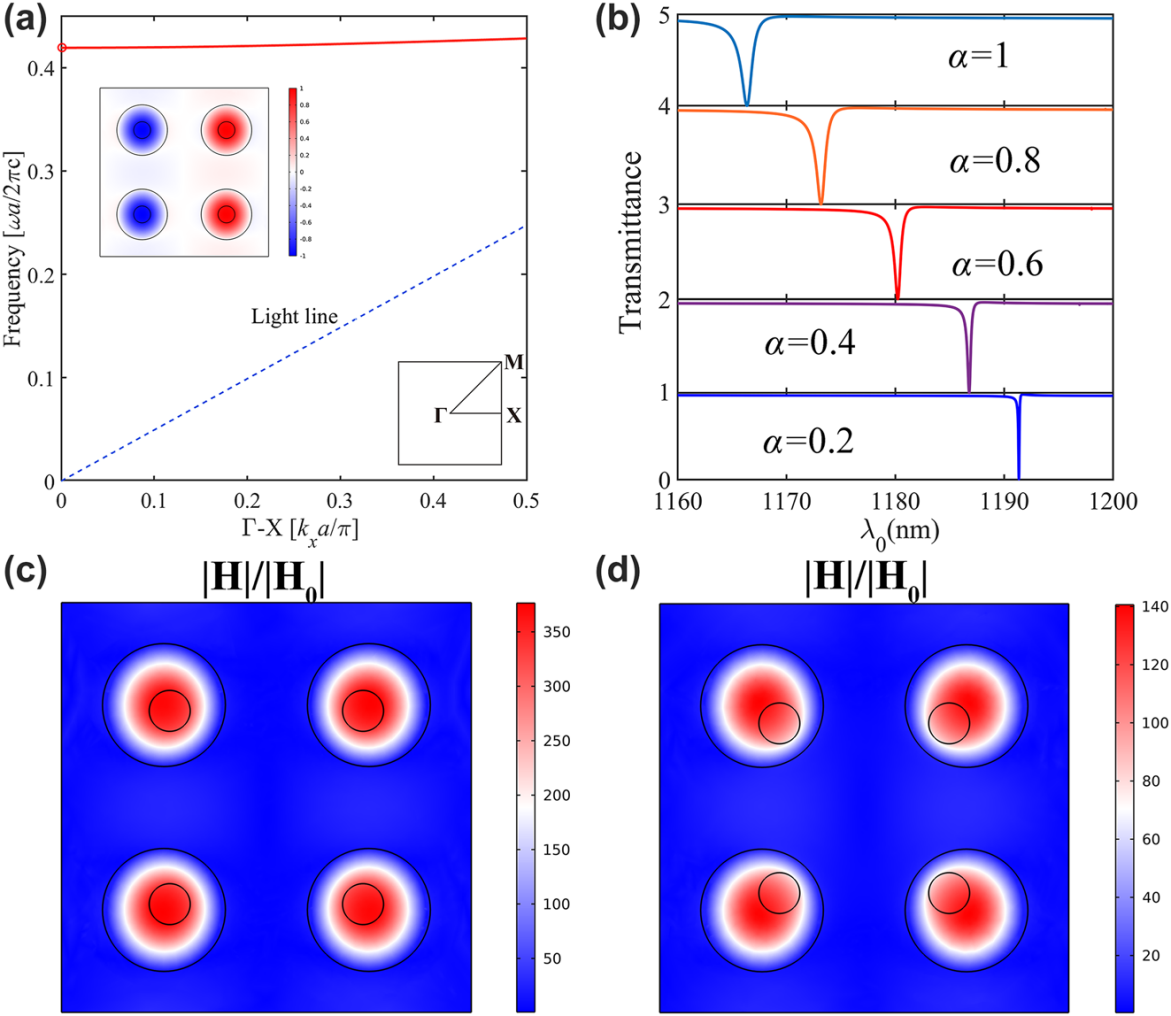
Characterization of Bound state in the continuum. (a) Simulated band structures at *α* = 0, circled in red is the BIC at the Γ point we want to study, the inset shows the magnetic field distribution. (b) Evolution of the transmission spectra versus *α*. (c) and (d) Magnetic field distribution on a plane passing through the middle of the metasurface at *α* = 0.2, 0.6 respectively.

According to the description above, we set the metasurface depicted in Figure 1(c) with geometry parameters *p* = 500 nm, *R* = 150 nm, *r* = 50 nm, *H* = 200 nm, and *h* = 100 nm. The refractive index of the Si is set as 3.46 [1], and χ (3) = 2.8e−18 m^2^/V^2^. The metasurface is placed in the air with a refractive index of *n* = 1. The finite element method (FEM) is utilized to calculate the linear transmission spectrum by applying periodic conditions in both the *x* and *y* directions of the supercell. The dispersion of eigenmodes of the metasurface is shown in Figure 2(a). Since the C_4ν_ symmetry of the structure, the BIC mode is polarization independent. The mode we discussed at the Γ point has been marked with a red circle. The blue dotted line is a light-line. At the highly symmetric case when *δ* = 0, the eigenmode’s electric field and magnetic field at Γ point are depicted in the inset of Figure 2(a), indicating the existence of BIC modes. Next, we calculated the transmission spectra for different asymmetric factors shown in Figure 2(b). Clearly, for *α* = 0 (Δ*d* = 0 nm), no transmission dips can be observed, and the linewidth vanishes. As *α* continues to increase, the linewidth of transmission spectra increases, and the resonant wavelengths have a blueshift. The q-BIC associated with the non-radiative Fano resonance mode is intrinsically accompanied by a giant field concentration in the resonators. Figure 2(c) and (d) illustrate the magnetic (|*H*|) field distributions at the peak wavelength (1191.3 nm), (1180.2 nm) identified in Figure 2(a) for the case *α* = 0.2, *α* = 0.6, respectively. The maximum field concentration occurs in the metasurface, a magnetic field enhancement factor larger than 380 is identified within the cylindrical meta-atoms with a hole at *α* = 0.2, and at *α* = 0.6, the magnetic field enhancement factor is about 140. Consistent with the trend of decreasing *Q*-factor upon increasing *α*, the magnetic field enhancement factor showed a significant drop.

## 3 Multipole analysis of q-BIC

**Figure 3:**
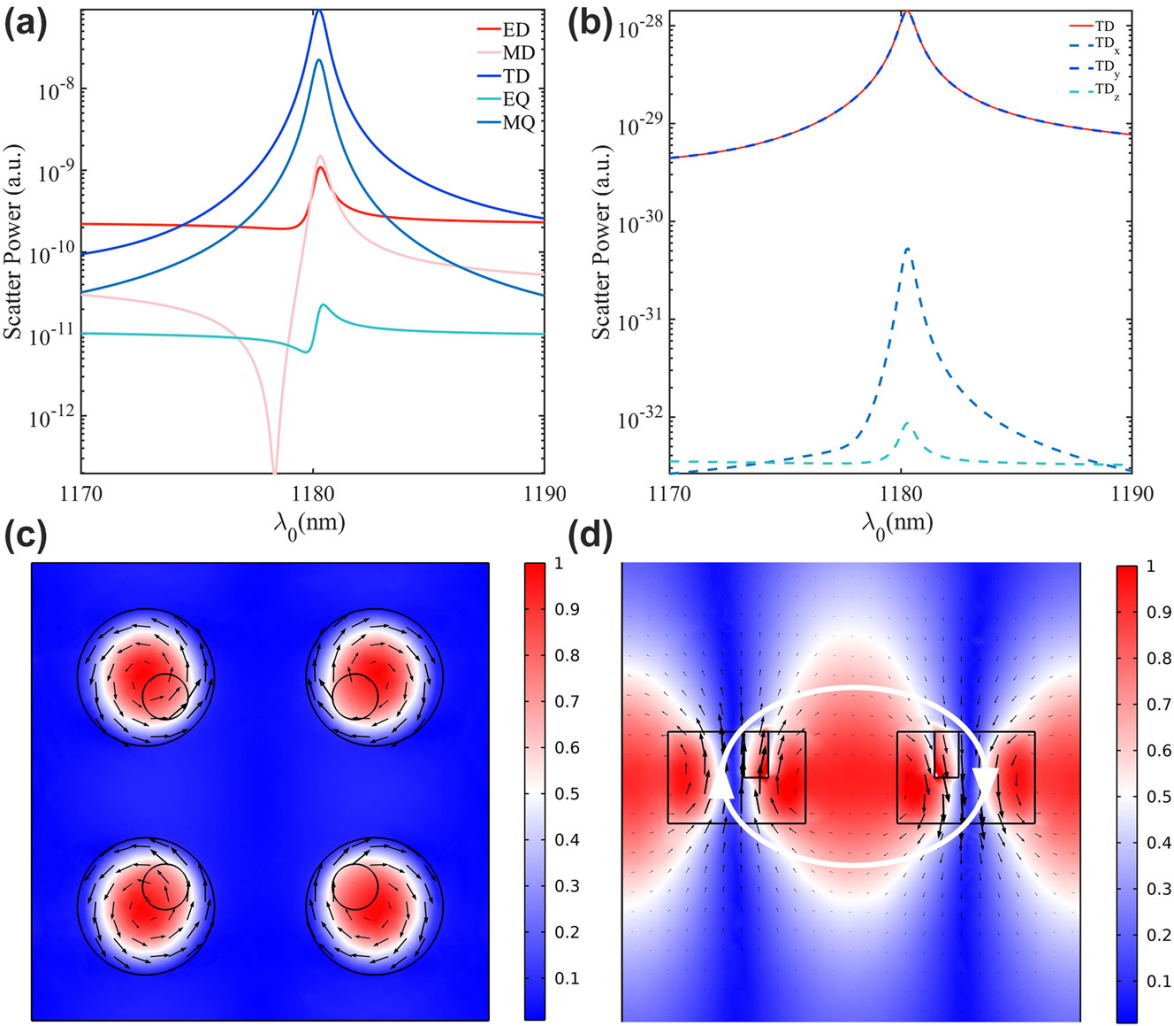
Scattered powers of the q-BIC mode at *α* = 0.6 from (a) multipoles and (b) *x*, *y*, and *z* components of the TD. (c) and (d) Electromagnetic field distribution of the q-BIC mode at *α* = 0.6.

To further figure out the mechanism of the BIC mode, the scattering powers of different multipoles, including the electric dipole (ED), the magnetic dipole (MD), the toroidal dipole (TD), the electric quadrupole (EQ), and the magnetic quadrupole (MQ) in the Cartesian coordinate system were analyzed and demonstrated in Figure 3(a) and (b). As seen in Figure 3(a), the contribution of the TD (deep blue solid line) is predominant, indicating that the BIC mode is mainly induced by the TD. By decomposing the *x*, *y*, and *z* components of the TD scattering power in Figure 3(b), we find that the y component of the scattering power from the TD dominates the TD scattering power dissipation and is almost equal to it in value. And compared to the *y* component, the *x* and *z* components of the scattered power of the TD are almost close to 0. The distribution of the normalized electromagnetic field for the BIC mode of the metasurface is presented in Figure 3(c) and (d). The black arrow represents the displacement current density and magnetic field vector in Figure 3(c) and (d), respectively. For BIC Mode, as shown in Figure 3(c), circulation orientations of displacement currents in the *y* direction of the Si neighboring cylindrical meta-atoms are opposite and the same in the *x* direction of the neighboring cylindrical meta-atoms, which indicates that the opposite phase magnetic dipoles along the *z* direction are induced in the *y* direction meta-atoms pair. Figure 3(d) depicts the magnetic field distribution at the *x*–*z* plane. As indicated by the white circle, opposite-phase magnetic dipoles form a closed magnetic vortex in the *x*–*z* plane. The magnetic field vector circulates clockwise between adjacent cuboids in the intra-cluster *x* direction meta-atoms and counterclockwise between adjacent cuboids in the inter-cluster *x* direction neighboring meta-atoms. The magnetic field distribution is head to tail, which is characteristic of TD multipole.

## 4 Free space nonreciprocal transmission and Bistability

**Figure 4:**
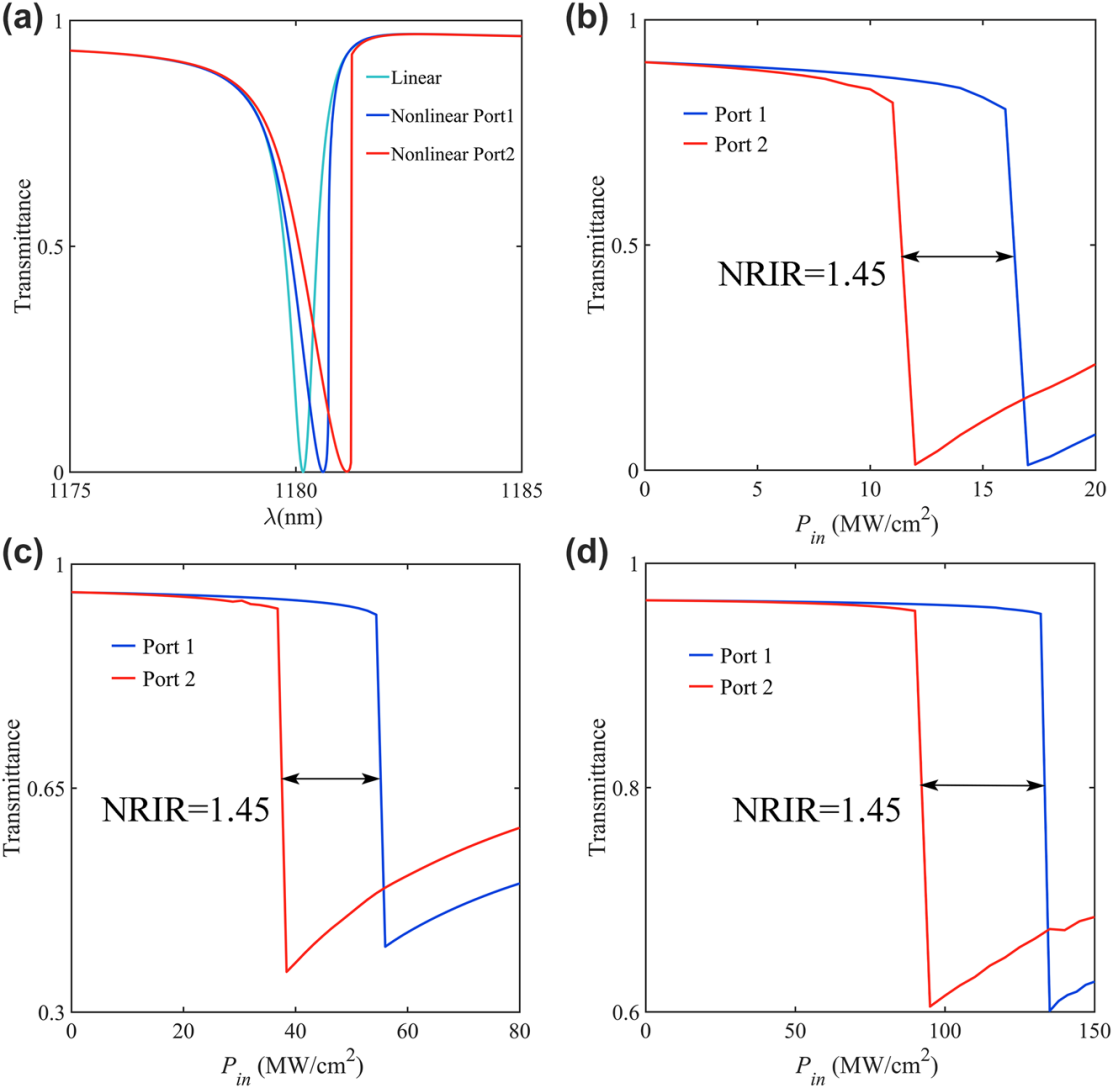
Passive nonreciprocal transmission. (a) Linear response and desired nonlinear response for which a transmission maximum from port 1 is aligned in terms of input intensity with a zero from port 2. (b–d) The intensity-dependent transmission at different excitation wavelengths.

To implement the nonreciprocal transmission in the proposed polarization-independent metasurface, we further perform nonlinear optical simulations using the FEM method in the frequency domain. In order to achieve optical isolation, the out-of-plane asymmetry is particularly important. We agree that the light from the +*z* direction to the −*z* direction is incident in the forward direction (port 1), and the opposite direction is incident in the back direction(port 2). Figure 4(a) shows the transmittance spectra at *α* = 0.6 when illuminated from each port with an incident intensity of 40 MW/cm^2^. The green line in Figure 4(a) displays the linear response of the structure, with a Fano resonant signature stemming from the superposition of a high-*Q* resonance in the dielectric atoms and a low-*Q* background resonance at the air-metasurface interfaces. According to the Kerr nonlinear effect, different field enhancements will lead to different refractive indices and thus give rise to the shift of resonant frequency. Since the resonator is out-of-plane asymmetric, this shift is distinct for excitation from opposite sides, enabling free space nonreciprocal transmission. Figure 4(a) reveals this mechanism by comparing linear and nonlinear responses at the same power from opposite sides.

Next, we consider the calculated nonlinear response of the metasurface, which is the same as Figure 4(a), presented in Figure 4(b)–(d). Figure 4(b)–(d) show the transmittance versus input power for three excitation wavelengths. Analogous to a p-n junction diode, the solid curves in Figure 4(b)–(d) represent effective optical I–V curves for light with a wavelength of *λ* = 1181 nm, *λ* = 1181.5 nm, *λ* = 1182 nm, respectively. For Figure 4(b), in the linear regime, when the incident power is relatively low, light is highly transmitted on both sides of the metasurface. As the incident power increases to 12 MW/cm^2^, the transmission intensity drops sharply in the backward direction (blue curve) while remaining very high in the forward direction (red curve), and the unidirectional transmission persists until around 17.4 MW/cm^2^. And here, the nonreciprocal intensity range (NRIR) is defined as the ratio of intensities from opposite directions for which transmission experiences a fast transition [1]. In fact, NRIR is closely related to the out-of-plane asymmetry of the structure, while having no relationship with excitation wavelengths. For Figure 4(c) and (d), the simulated intensity-dependent port-to-port transmission for excitation for another two different excitation wavelengths from Figure 4(b) explains the mechanism that NRIR is excitation wavelength independent. For any excitation wavelength and transmission direction, the minimum transmission intensity in the nonlinear response is dependent on the linear transmission spectra (Figure 4(a)), the different transmission intensities are shown in Figure 4(b)–(d) for different excitation wavelengths. The transmittance drops to almost zero with the increasing incident power for Figure 4(b), but as shown in Figure 4(c) and (d), there is no way to get to zero for the minimum transmittance.

In particular, the trade-off for the maximum nonlinear forward transmission and the NRIR, can be explained by the temporal coupled mode theory(TCMT). In the context of TCMT, a single resonance with 2 ports is expressed as

$$\frac{\text{d}a}{dt}=\left(-j\omega_{0}-\gamma_{1}-\gamma_{2}-\gamma_{v}\right)a+\left(k_{1}\;\;k_{2}\right)\left(\begin{array}{c} s_{1+}\\ s_{2+} \end{array}\right),$$

(1) $$\left(\begin{array}{c} s_{1-}\\ s_{2-} \end{array}\right)=C\left(\begin{array}{c} s_{1+}\\ s_{2+} \end{array}\right)+\left(\begin{array}{c} k_{1}\\ k_{2} \end{array}\right)a,$$

$$C=\left(\begin{array}{cc} r_{B} & -jt_{B}\\ -jt_{B} & r_{B} \end{array}\right)$$

Here, *a* is the resonance amplitude, *ω*_0_ is the resonance frequency, *γ*_*v*_ is the intrinsic cavity loss rate and *γ*_*v*_ = 0 since we do not consider losses at this point, *γ*_in_ = *γ*_1_ + *γ*_2_ is the cavity decay rate due to coupling into the two ports, with decay rates *γ*_1_ and *γ*_2_, respectively, *s*_1+_ and *s*_2+_ are the amplitudes of the incoming waves from the two ports, *s*_1−_ and *s*_2−_ are the amplitudes of the outgoing waves, and *k*_1_, *k*_2_ are the complex coupling coefficients between the ports and the resonance. The scattering matrix *C* represents the direct coupling between incoming and outgoing waves, with *r*_*B*_ and *t*_*B*_ being the corresponding amplitude reflection and transmission coefficients, which satisfy $r_{B}^{2}+t_{B}^{2}=1$ .

According to the energy conservation and time-reversal symmetry property of the cavity, the conditions can be obtained as follows,

(2) $$k_{1}=\sqrt{2\gamma_{1}}{\text{e}}^{j\theta_{1}},\;k_{2}=\sqrt{2\gamma_{2}}{\text{e}}^{j\theta_{2}},$$

(3) $$\left(\begin{array}{cc} r_{B} & it_{B}\\ it_{B} & r_{B} \end{array}\right)\left(\begin{array}{c} k_{1}^{*}\\ k_{2}^{*} \end{array}\right)=-\left(\begin{array}{c} k_{1}\\ k_{2} \end{array}\right).$$

Through the temporal coupled mode theory and the conditions, the transmission coefficient of the system can be derived easily as

(4) $$T={\left|t_{B}\right|}^{2}\frac{{\left(x\mp x_{0}\right)}^{2}}{x^{2}+1}.$$

Here, *x* = (*ω*_0_ − *ω*)/*γ* is the detuning factor of the resonator, and *x*_0_ = $\sqrt{\frac{4\gamma_{1}\gamma_{2}}{\left[T_{bg}{\left(\gamma_{1}+\gamma_{2}\right)}^{2}\right]}-1}$ is a characteristic parameter of the resonator that provides the detuning from the resonance frequency at which transmission is zero. The positive (negative) sign in Eq. (3) corresponds to the case where the frequency of the transmission zero is higher (lower) than the resonant frequency. If we define *κ* = |*k*_1_|^2^/|*k*_2_|^2^ = *γ*_1_/*γ*_2_ as the asymmetry factor from different ports, Eq. (3) can be rewritten as

(5) $$T=\frac{4\kappa }{{\left(\kappa +1\right)}^{2}}\frac{{\left(x\mp x_{0}\right)}^{2}}{\left(x^{2}+1\right)\left(x_{0}^{2}+1\right)}.$$

Considering that for a symmetric Fano resonator maximum transmission is unitary, we know that asymmetry imposes the following bound on the transmission of Fano resonators when the resonator is symmetric, *κ* = 1:

(6) $$T\leq \frac{4\kappa }{{\left(\kappa +1\right)}^{2}}.$$

It can be shown that the NRIR is always equal to the linear electromagnetic asymmetry, NRIR = *Κ* = |*E*_1_|^2^/|*E*_2_|^2^, thus,

(7) $$T_{\max}^{\text{nonlinear}\frac{4\text{NRIR}}{\text{NRI}{\text{R}}^{2}+1}}$$

Therefore, although increasing the out-of-plane asymmetry of the structure can obtain an immense NRIR value, the cost is lower in transmission intensity.

Next, we describe the mechanism of nonlinear bistability. If we assume the resonators to be nonlinear, the nonlinearity results in a shift of the resonance frequency of the resonator as

(8) $$\omega_{0}=\omega_{0,\text{lin}}\left(1-\frac{|a{|}^{2}}{{\left|a_{0}\right|}^{2}}\right)$$

where *ω*_0,lin_ is the resonance frequency in the linear regime and |*a*_0_|^2^ is the energy in the nonlinear cavity. Considering that the nonlinear isolator is excited from the *i*_th_ port with a monochromatic signal *s*_*i+*_ at frequency *ω*, we get the equation as follows,

(9) $$\left(1-\frac{\omega_{0}}{\omega_{0,\text{lin}}}\right)\left[{\left(\omega -\omega_{0}\right)}^{2}+\gamma^{2}\right]=\frac{{\left|k_{i}\right|}^{2}P_{i}^{\text{in}}}{{\left|a_{0}\right|}^{2}}.$$

Here, $P_{i}^{\text{in}}$ = |*s*_*i+*_|^2^, *γ* and *k*_*i*_ are the same as in the linear regime, *ω*_0_ is the input intensity.

From this equation, we can understand an important mechanism in nonlinear optical cavities. For different input light intensities, there will be different resonance frequency offsets and the overall response of Fano nonlinear isolators can be adjusted by the factor *κ* = |*k*_*1*_|^2^/|*k*_*2*_|^2^ for different incident ports. So that the NRIR of nonreciprocal response could be largely tuned and the bistability phenomenon can be obtained.

Therefore, although increasing the out-of-plane asymmetry of the structure can obtain an immense NRIR value, the cost is lower in transmission intensity.

According to the discussion in the previous article, we will again analyze the influence of in-plane asymmetry and out-plane asymmetry on nonreciprocal transmission. The in-plane asymmetry is usually utilized to transform the true BICs to q-BICs since true BICs possess an infinite *Q*-factor, zero linewidth, and cannot be excited. We can adjust the degree of in-plane asymmetry *α* to tune the *Q*-factor of the metasurface and maximize the nonlinear interactions, but it is not necessary to achieve nonreciprocity. Instead, the out-plane symmetry is vital for nonreciprocal behavior along the +*z* and −*z* directions. By controlling the height *H* and *h*, we can adjust the electromagnetic asymmetry of the structure and the value of NRIR.

**Figure 5:**
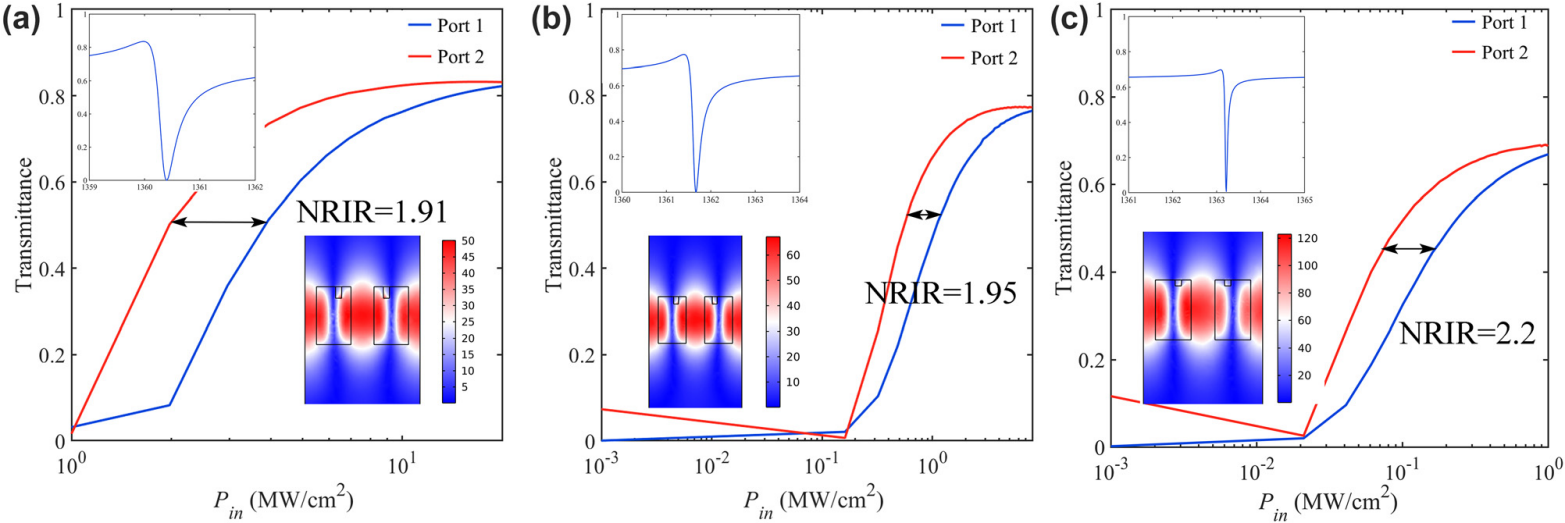
Nonlinear characterization with different values of *H*, *h*: (a) *H* = 500 nm, *h* = 100 nm, (b) *H* = 500 nm, *h* = 80 nm, (c) *H* = 500 nm, *h* = 50 nm. In each panel, the insets above show linear transmission spectra of three devices, the insets below display the electric field enhancement.

Next, we keep other parameters unchanged and adjust the height of the cylindrical meta-atoms H and the height of the etch cylindrical hole h to confirm the effect of out-of-plane asymmetry on NRIR. The intensity-dependent transmission of three representative metasurfaces is shown in Figure 5(a–c) with out-of-plane asymmetries ranging from values (*H* = 500 nm, *h* = 100 nm, Figure 5(a), *H* = 500 nm, *h* = 80 nm, Figure 5(b), and *H* = 500 nm, *h* = 50 nm, Figure 5(c)). In each panel, the insets show the linear transmission spectra and a vast field enhancement of the electric field, respectively. As anticipated, the device with the most enormous out-of-plane asymmetry (Figure 5(c), *H* = 500 nm, *h* = 50 nm) also features the broadest nonreciprocal intensity range (NRIR = 2.2), while its maximum transmission in the forward direction is limited to ∼ 0.7. Strong field enhancement can result in nonreciprocal transmission at lower power, while different out-of-plane asymmetries will lead to different transmission levels and NRIR. Consistent with the previous, the minimum and maximum transmission obtained in the nonlinear response match the minimum and maximum transmission in the corresponding linear transmission spectrum. When we reduce the out-of-plane asymmetry, the corresponding forward maximum transmission efficiency will increase, while the NRIR shrinks.

**Figure 6:**
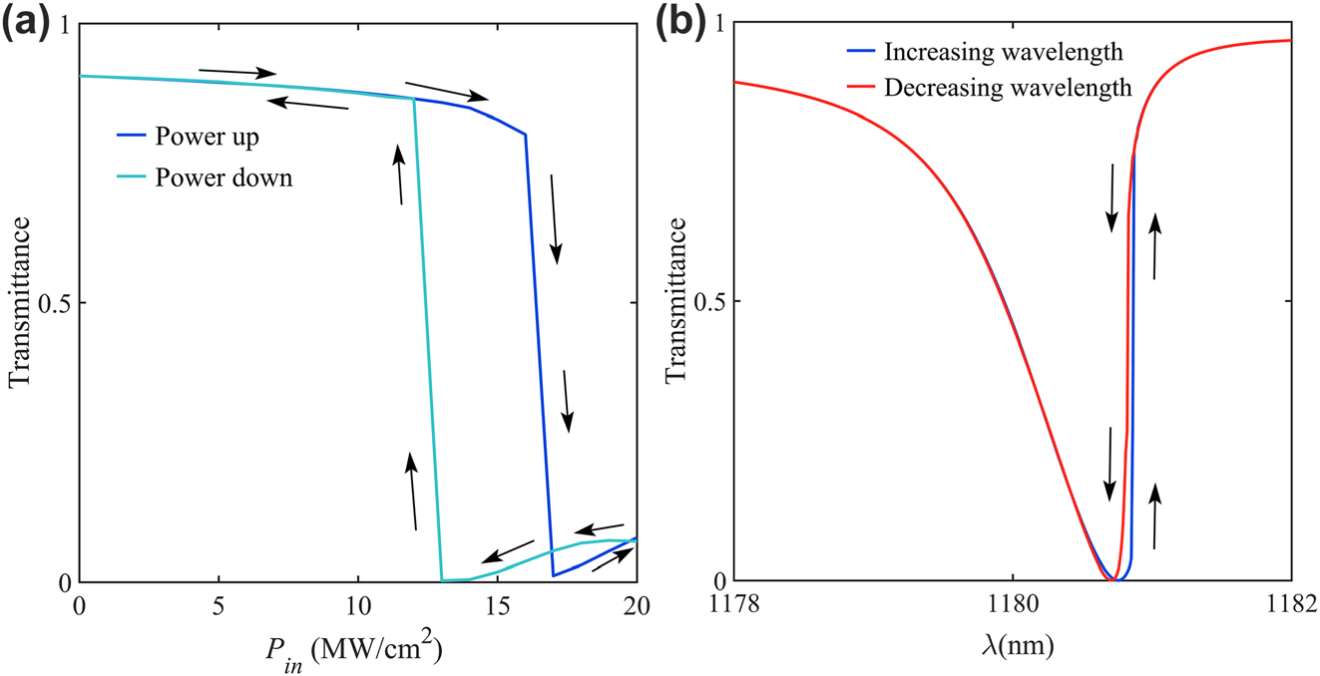
Optical bistability. (a) Measured the forward transmission of the metasurface by scanning the power up (blue curve) and down (cyan curve). (b) Measured the forward transmission of the metasurface by Scanning with increasing wavelength (blue curve) and decreasing wavelength (red curve).

We obtain the intensity-dependent transmission while sweeping the power up(blue curve) and down(green curve) at *H* = 200 nm, *h* = 100 nm, shown in Figure 6(a), and calculate the wavelength-dependent transmission with the wavelength scanned from short to long wavelength and then also from long wavelength to short shown in Figure 6(b). For Figure 6(a), because of the existence of third-order nonlinearity, the bistable phenomenon can be predicted. There will be two stable states in the case of the same incident power. For power up, the transmission efficiency suffers a sudden drop at an intensity of about *I*_peak_ = 17 MW/cm^2^ with increasing intensities, corresponding to the transition from the one stable state, which has a high transmittance, to another one which has low transmittance. Then as the incident power gradually decreases from high power, the system remains in the stable state, which is transmitted with less efficiency until the peak intensity is about *I*_peak_ = 12 MW/cm^2^, after which it jumps back to another steady state which is transmitted with more efficiency. For Figure 6(b), we scan the transmittance with increasing wavelength (red curve) and decreasing wavelength (blue curve), respectively. Since the initial field condition of different wavelengths, the various Kerr shifts take place in different ways of scanning wavelengths. All nonlinear simulations in the above were performed with a wavelength-increasing scan.

## 5 Conclusions

In conclusion, we present the design and analysis of a polarization-independent nonreciprocal metasurface with C_4ν_ symmetry, based on symmetry-protected BIC resonances. The *Q*-factor and linewidth of q-BICs were adjusted by manipulating the distance between the etched hole and the center *O* of the metasurface. Our investigation revealed that the toroidal dipole was the main contributor to the mechanism of symmetry-protected BIC mode. We observed that the scattering power from the *y* component of the toroidal dipole was dominant. Exploiting the nonlinear properties of the material in combination with the out-of-plane asymmetry of the metasurface structure, we achieved free space nonreciprocal transmission. Our study also highlights the ability to tune the nonreciprocal intensity range by controlling the structure parameters. We discuss the trade-off between the maximum nonlinear forward transmission and the nonreciprocal intensity range. Additionally, we observe the bistable phenomena of the nonreciprocal system. Our research demonstrates a robust and widely applicable approach for obtaining free-space fully-passive nonreciprocal propagation by leveraging q-BICs and material nonlinearities. Our findings pave the way for various applications, including the protection of high-power lasers and nonreciprocal signal routing for analog and quantum computing.
